# Repeated social defeat promotes persistent inflammatory changes in splenic myeloid cells; decreased expression of β-arrestin-2 (ARRB2) and increased expression of interleukin-6 (IL-6)

**DOI:** 10.1186/s12868-020-00574-4

**Published:** 2020-05-29

**Authors:** Dhaksshaginy Rajalingam, Ingeborg Nymoen, Daniel Pitz Jacobsen, Mina Baarnes Eriksen, Erik Dissen, Morten Birkeland Nielsen, Ståle Valvatne Einarsen, Johannes Gjerstad

**Affiliations:** 1 Department of Psychosocial Science, University of Bergen, Bergen, Norway; 2 National Institute of Occupational Health, Oslo, Norway; 3 Institute of Basic Medical Sciences, University of Oslo, Oslo, Norway

**Keywords:** Social stressors, Repeated social defeat, Bullying, ADRB2, ARRB2, IL-6

## Abstract

**Background:**

Previous studies suggest that persistent exposure to social stress in mammals may be associated with multiple physiological effects. Here, we examine the effects of social stress in rats, i.e. repeated social defeat, on behavior, hypothalamic–pituitary–adrenal (HPA)-axis and immune system.

**Methods:**

A resident-intruder paradigm, where an intruder rat was exposed to social stress by a dominant resident rat for 1 hour each day for 7 consecutive days was used. The day after the last stress exposure in the paradigm the data were analyzed. Variation in social interaction was observed manually, whereas locomotion was analyzed off-line by a purpose-made software. Gene expression in the pituitary gland, adrenal gland and myeloid cells isolated from the spleen was measured by qPCR.

**Results:**

The exposure to social stress induced decreased weight gain and increased locomotion. An increased nuclear receptor subfamily group C number 1 (NR3C1) expression in the pituitary gland was also shown. In myeloid cells harvested from the spleen, we observed decreased expression of the β_2_-adrenergic receptor (ADRB2) and β-arrestin-2 (ARRB2), but increased expression of interleukin-6 (IL-6). Subsequent analyses in the same cells showed that ARRB2 was negatively correlated with IL-6 following the stress exposure.

**Conclusion:**

Our results show that that the experience of social stress in the form of repeated social defeat in rats is a potent stressor that in myeloid cells in the spleen promotes persistent inflammatory changes. Future research is needed to examine whether similar inflammatory changes also can explain the impact of social stress, such as bullying and harassment, among humans.

## Background

Previous studies show that environmental stressors in mammals induce increased activity of the sympathetic nervous system (SNS) and the hypothalamic-pituitary-adrenal (HPA)-axis [1–3]. Activation of these systems may be associated with altered behavior [4], hormonal signaling [5] as well as changes in the immune system, for review see [6]. Environmental stressors may promote myelopoiesis in the bone marrow [7], glucocorticoid (GC) resistance in the brain and the spleen [8], increased circulatory levels of cytokines [9] and altered inflammatory profile in the brain [10].

Recent data demonstrate that exposure to repeated social defeat—through the resident-intruder paradigm in rats or bullying in humans—is a strong environmental stressor [11]. Previous observations show that environmental stressors may induce neuronal activation of the reticular formation in the brain stem including locus coeruleus (LC) [1], which in turn affects efferent sympathetic nerve fibers that innervate the adrenal gland and spleen [12, 13]. In the adrenal medulla, this innervation results in epinephrine (E) release from chromaffin cells into the circulation [14, 15]. In addition, exposure to environmental stressors leads to activation of the paraventricular nucleus (PVN) of the hypothalamus [1]. This stimulates the HPA-axis through corticotropin releasing hormone (CRH) [16] that promotes secretion of corticosteroids (CORTs) from the adrenal cortex [17].

Regarding the autonomic influence on the thymus, spleen, lymph nodes and bone marrow, norepinephrine (NE) signaling by efferent sympathetic nerve fibers plays a crucial role [18]. Stimulation of adrenergic receptors on immune cells causes changes in differentiation, inflammatory profile and migration capacity [19, 20]. For instance, increased sympathetic signaling may facilitate the induction of genes involved in myeloid lineage effector functions, signal transduction and transcription control [7]. Earlier observations suggest that stress-induced inflammation and myelopoiesis may be linked to increased activity of transcription factors such as nuclear factor kappa B (NF-ĸB) [7, 9].

Evidence exists that activation of the SNS and HPA-axis may be involved in the regulation of leukocyte trafficking [21, 22]. Leukocyte counts have also revealed increased cell numbers in bone marrow, peripheral blood and spleen following repeated social defeat in mice [23]. These observations are consistent with stress-induced changes in splenic neutrophil and macrophage numbers. Moreover, stress may cause leukocyte recruitment from the bone marrow to the spleen [23]. Increased levels of granulocyte-monocyte colony-stimulating factor (GM-CSF) in circulating monocytes may be a part of the underlying mechanism [7]. Further observations suggest that repeated social defeat may lead to increased release of monocyte chemoattractant protein-1 (MCP-1) from microglia cells [24], which induces monocyte recruitment from the spleen to the capillaries in the brain [10].

It has been proposed that environmental stressors, through activation of β2-adrenergic receptors (ADRB2s), may lead to inflammatory changes of splenic immune cells [7, 25, 26]. In addition, earlier observations suggest that persistent activation of ADRB2s in murine macrophages increases mRNA and protein levels of pro-inflammatory cytokines such as interleukin-6 (IL-6) and interleukin-1β (IL-1β) [27]. Moreover, evidence exists that β2-adrenergic signaling also involves β-arrestin 2 (ARRB2), a protein known to inhibit NF-ĸB nuclear translocation by stabilizing cytoplasmic IĸBα activity through ADRB2 activation [28, 29]. However, whether or not ADRB2 and ARRB2 may be associated with the expression of cytokines such as IL-6 during social stress has not been clarified. The aim of the present study was to examine the effect of social defeat on the neuroimmune interface.

## Methods

### Animals

As described below, a resident-intruder paradigm, where Sprague–Dawley intruder rats were exposed to social stress by dominant Long Evans resident rats; 1 h each day (between 09:00 and 13:00) for 7 consecutive days, was used to study stress-induced changes in the HPA-axis and the immune system. Each of the ten male Long Evans rats (500–550 g) was housed with a female Long Evans rat (200–250 g) in a 0.56 m^2^ cage. The ten male Sprague–Dawley rats (300–400 g) used as intruders were housed in pairs, as were the ten male Sprague–Dawley rats (300–400 g) used as controls. The different strains–Long Evans rats from Envigo; USA and Sprague–Dawley rats from Janvier Labs; France–were kept in separate rooms. All rats were acclimatized to a 12:12 h light:dark cycle, ventilation rate of 15 × air per hour, 21–22 °C and 45–55% humidity. At all times, the rats had ad libitum access to food and water. Bedding was changed once a week. All animal procedures were approved by the Norwegian Food Safety Authority and performed in conformity with laws and regulations controlling experiments and procedures on live animals in Norway.

### Screening

To ensure dominant behavior of Long Evan males i.e., the resident rats in the paradigm, a screening was performed prior to the stress-conditioning week. Top ten aggressive rats were chosen based on the highest incidences of attacks over a period of 10 min.

### Resident-intruder paradigm

First, the female rat was temporarily removed from the resident cage 1 hour before the stress conditioning. Next, the stress conditioning was performed by introducing the intruder animal into the resident cage. The male resident and intruder rat were separated upon three episodes of social defeat (submissive supine posture, freeze or flight), or after 10 min of interaction by a perforated plastic wall, allowing the intruder rat to still see, smell and hear the resident rat. Finally, after 60 min in the resident cage, the intruder rat was returned to its home cage, and the female rat was returned to the resident cage. The conditioning procedure described above was repeated for 7 days. To prevent habituation to the dominance establishment with the resident rat, the intruder animals were introduced to a new resident animal every day. The animals randomized to control followed the same procedure except that they visited a foreign cage without a resident rat.

### Social interaction test

A modified version of the social interaction test was used to assess the social interaction behavior of the Sprague–Dawley rats (i.e., the test rats) following 1week of stress or control conditioning [30]. More specifically, the social interaction test was conducted 1 day after the last episode of defeat. The test arena was a purpose made box (0.56 m^2^) divided into three compartments by two gated plastic walls and a small wire-like container in each flanking compartment. The test rats were allowed to habituate in the center compartment for 4 minutes (Additional file 1: Fig. S1a) before a novel rat of the same strain was placed into one of the small wire-like containers (Additional file 1: Fig. S1b). The subsequent opening of the gates allowed the test rat to move freely between the compartments for 6 minutes (Additional file 1: Fig. S1c). Movement and behavior of the test animals were recorded by a camera placed in a rack above the box. Thus, changes in behavior were examined after the experiments, including the time spent in each chamber and the time spent in direct social interaction with the novel rat. The novel rats were habituated to the wire-like container prior to the social interaction test, but did not take part in the resident-intruder paradigm.

### Video analysis

Recorded videotapes of rats moving in the three-chamber box were analyzed using a purpose-made software program, which was programmed and developed in C. The time spent in each of the three chambers and locomotion of rats (10 s intervals) were scored by the software.

### Anesthesia and blood sampling

Following the social interaction test and one hour rest in their home cage, on day 8, the intruder Sprague–Dawley and control rats were sedated with 5% isoflurane in air in a gas box prior to being moved to a 3% isoflurane anesthetic gas mask. Absence of withdrawal reflexes was considered sufficient anesthesia for surgery.

The animal was fixated in a dorsal recumbence position and a v-cut through the skin and abdominal wall was made. The heart was exposed by opening the thoracic cage, cutting through the diaphragm. A 10 mL syringe with a 1.2 mm cannula coated with 1.8 mg/mL EDTA (Sigma Life Science; Switzerland), was inserted into the left ventricle (cardiac puncture). Blood samples of 2 ml were drawn from the exposed and control Sprague–Dawley rats. In accordance with the procedure previously described, 500 µL of the blood was immediately placed on liquid nitrogen for NE and CORT concentration measurements performed (Additional file 1: Fig. S2) [31].

### Tissue harvesting

All Sprague–Dawley rats were euthanized by dislocation of the neck under isoflurane anesthesia. The pituitary gland and adrenal glands were harvested, frozen on liquid nitrogen and later stored in a −80 °C freezer.

### Enrichment of splenic myeloid cells

The spleen was mechanically disrupted with scissors, and pieces of spleen tissue were passed multiple times through a 10 mL syringe and filtered through a 70 µM cell strainer in order to get a single cell suspension. Mononuclear cells were retrieved by density centrifugation. The suspension was diluted with PBS (GE Healthcare Lifesciences; USA), loaded on top of a 15 mL Lymphoprep^TM^ medium (STEMCELL technologies; Norway) and centrifugated (400x*g* for 30 min at 4 °C). The layer of mononuclear cells was carefully aspirated, diluted in PBS supplemented with 2% FBS, washed by centrifugation (300x*g*, 10 min, 4 °C) and resuspended in PBS (2% FBS). Myeloid cells were purified from the spleen mononuclear fraction by immunomagnetic bead separation. To avoid unspecific monoclonal antibody (mAb) binding, the Fc receptors were pre-blocked by incubating the cells in PBS with 10% rat serum for 15 min at 4 °C. Subsequently, cells were incubated with a biotinylated mouse mAb (OX41) specific for rat CD172a (SIRP-α, expressed on the surface of all myeloid cells) at 2 µg/mL in PBS (10% rat serum) for 15 min at 4 °C and washed three times in PBS (2% FBS, 10 mM NaN_3_) before incubation with streptavidine-coated magnetic microbeads (MACS, Miltenyi Biotec; Germany) resuspended in PBS supplemented with 2 mM EDTA and 0.5% BSA for 30 min at 4 °C, using 40 µL of beads per 4 x 10^7^ cells. The cells were then run through MACS LS columns in the magnetic field of a quadroMACS^TM^ separator (Miltenyi Biotec; Germany) to separate bead-captured cells from unstained, non-myeloid cells according to manufacturer instructions.

### Flow cytometry

Flow cytometry was used to verify the enrichment of CD172 positive cells and the nature of contaminating non-myeloid cells. Two separate mixes of fluorochrome-conjugated mAbs for test and isotype controls were used, diluted in PBS (2% FBS, 10 mM NaN_3_) (Additional file 1: Table S1). Staining with isotype control antibodies was included to evaluate unspecific mAb binding capacity to splenic cell subsets.

A small fraction of the cell sample i.e., 3 x 10 ^5^, was used for flow cytometry analysis and incubated with 50 µL mAb test or isotype mix (2 µg/mL) in PBS (2% FBS, 10 mM NaN_3_) for 30 min on ice. After staining with primary antibody mixes, the cells were washed three times by centrifugation (300x*g*, 2 min, 4 °C), resuspended in PBS (2% FBS, 10 mM NaN_3_) and incubated with Streptavidin-Alexa Fluor 647 conjugated for detection of OX41-biotin or IgG1-biotin binding, respectively. Cells were washed and analyzed on a CytoFlex flow cytometry (Beckman Coulter Life Sciences, USA) using CytExpert software.

### RNA isolation and cDNA synthesis

The allprep DNA/RNA/miRNA Universal Kit (Qiagen; Germany) was used to isolate total RNA from the frozen pituitary, adrenal and enriched myeloid cells. Total RNA was extracted by homogenizing the frozen tissue with magnetic beads in a bead beater. The lysate was then used for RNA isolation following the manufacturer’s protocol. Synthesis of cDNA from these tissues was carried out using the qSCript cDNA synthesis kit (Quanta Biosciences Inc.; USA).

### Gene expression analyses

RNA quantification of the different genes was achieved by a two-step real-time reverse transcription qPCR (RT-qPCR). Primer sequences (fwd,rev) were from Sigma Life Sciences, Switzerland: POMC (5′AACGCCATCAAGAAC3′ and 5′AAGGTTTTATTTCCTAACTACAC3′); NR3C1 (5′CAGAGAATGTCTCTACCCTG3′ and 5′CTTAGGAACTGAGGAGAGAAG3′); MC2R (5′AGAAACTGGATCCTTCCG3′ and 5′TGGTGTGTTCATACGAATTG3′); β-actin (5′CTAAGGCCAACCGTGAAAAGA3′ and 5′ACAACACAGCCTGGATGGCAT3′); IL-6 (5′TGCCCTTCAGGAACA3′ and 5′AAGGCAGTGGCTGTC3′); ADRB2 (5′AAAGAGAGAGAGAGAGACT3′ and 5′ACAACACTTCAGACAGAAAC3′); HPRT (5′ACTGGTAAAACAATGCAGGAC3′ and 5′CCTGAAGTGCTCATTATAGTC3′); PtPrc (5′GCTATAAAAAGACCCCTTCAG3′ and 5′CATAGGCAAATAGAGACACTG3′); ARRB2 (5′GCAGCCAGGACCAGAGGACA3′ and 5′CCACGCTTCTCTCGGTTGTC3′). PCR was run on Quantstudio 5 (Thermofisher Scientific; Norway) and analyzed using Quantstudio^TM^ Design and Analysis Software.

### Statistics

The data were shown by representative examples and mean ± standard error of the mean. Statistical analyses were conducted with Sigmaplot 14.0 and the level of significance was set to p < 0.05. Shapiro–Wilk test was run to assess normality. Differences in body weight, social interaction, locomotion, gene expression levels and differences in percentage of myeloid cells between exposed group and control group were analyzed using Student’s *t* test.

## Results

### Behavior

**Fig. 1:**
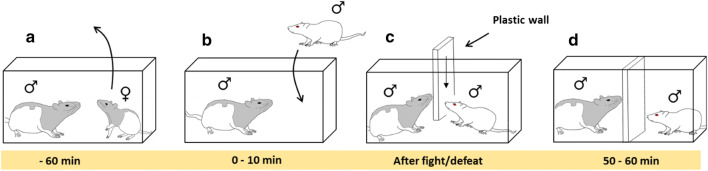
Resident-intruder paradigm. The set-up used to induce defeat stress in the intruder Sprague–Dawley rats (white) in the cage of the resident male Long Evans rat (black-hooded). **a** The Long Evans female was removed 60 min prior to the experiment. **b** An intruder Sprague–Dawley rat was placed in the home cage of a resident male Long Evans rat. **c** Upon three episodes of social defeat (submissive supine posture, freeze or flight), or after 10 min of interaction, a plexiglas wall was used to separate the resident and intruder rats. **d** Sensory interaction in the divided cage was allowed for the remaining time of the hour. Both the male Sprague–Dawley and female Long Evans rats were returned to their home cage after the conditioning. (Illustrated by Nymoen, I.)

**Fig. 2:**
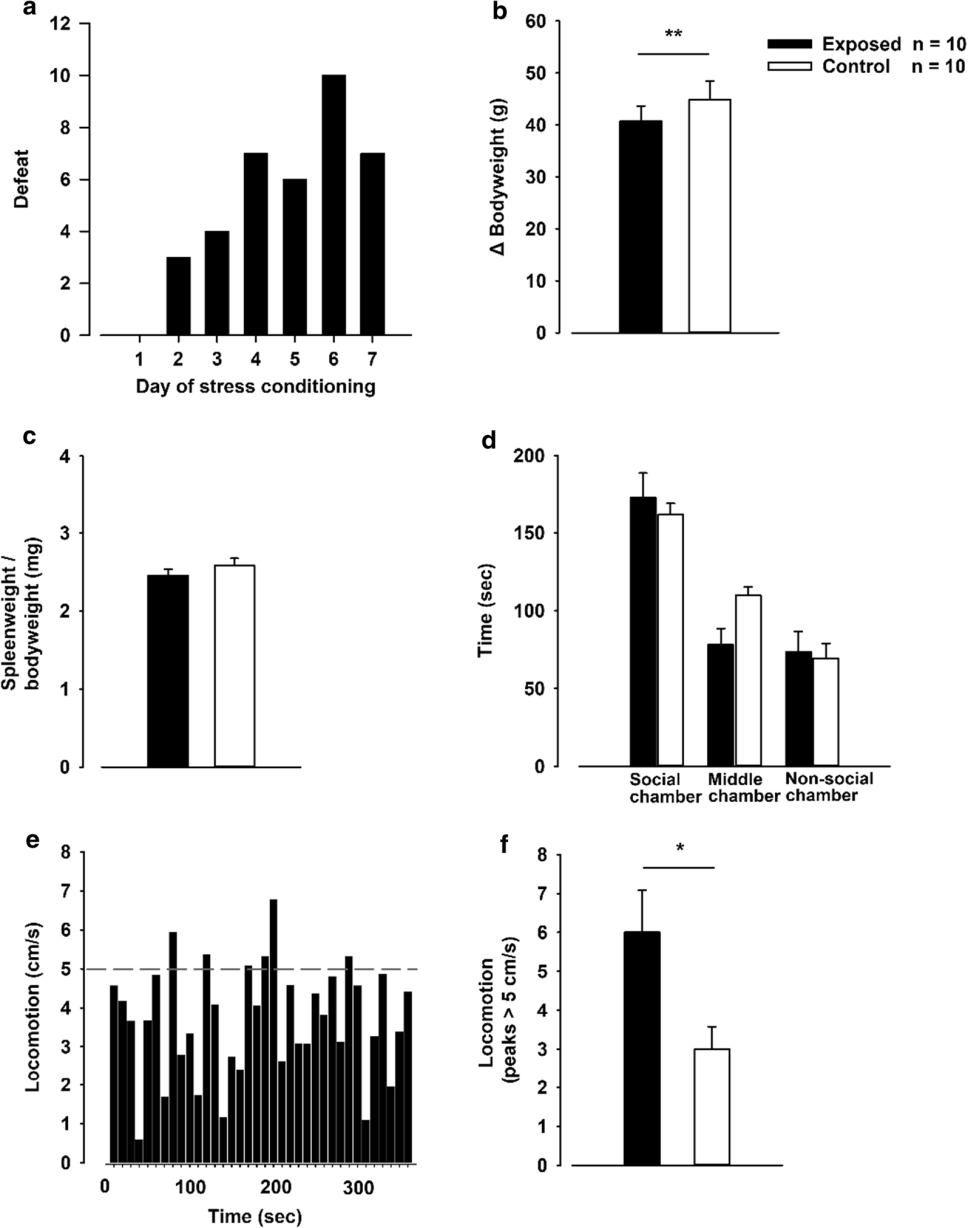
Behavior, weight gain and locomotion. **a** Number of defeated stress-exposed rats (intruder rats) during conditioning at day 1–7. **b** Bodyweight at day seven in stress-exposed rats versus control rats (relative to baseline), p = 0.007. **c** Organ-to-bodyweight ratio of the spleen in stress-exposed rats versus control rats. **d** Social interaction test; time spent in the three different chambers, stress-exposed rats versus control rats. **e** Example of locomotion in the three different chambers (10 s intervals). **f** Peak locomotion > (5 cm/s) stress-exposed rats versus control rats, p = 0.029. *p < 0.05, **p < 0.01, Students t-test

The resident-intruder paradigm changed the behavior of the intruder rats in the residential cage (Fig. 1). For each day passing, the number of rats showing subordinate defeat behavior increased. After 6 days of stress conditioning all intruder rats showed a clear social defeat within the 10-min frame (Fig. 2a).

The exposed rats gained less weight during the conditioning week, compared to controls (Fig. 2b). However, we did not observe any increase of spleen weight-to-bodyweight ratio (Fig. 2c), and thus there was no evidence of splenomegaly following stress exposure.

Following the conditioning week, all exposed and control animals went through a social interaction test. No difference was observed between the two groups, evaluated by time spent in the three different chambers (Fig. 2d). Locomotion (cm/s) of the rats in 10 s intervals was measured by a computer. The stress exposed rats had significantly higher locomotion compared to control rats (Fig. 2e, f).

### HPA-axis gene expression and NE/CORT in plasma

**Fig. 3:**
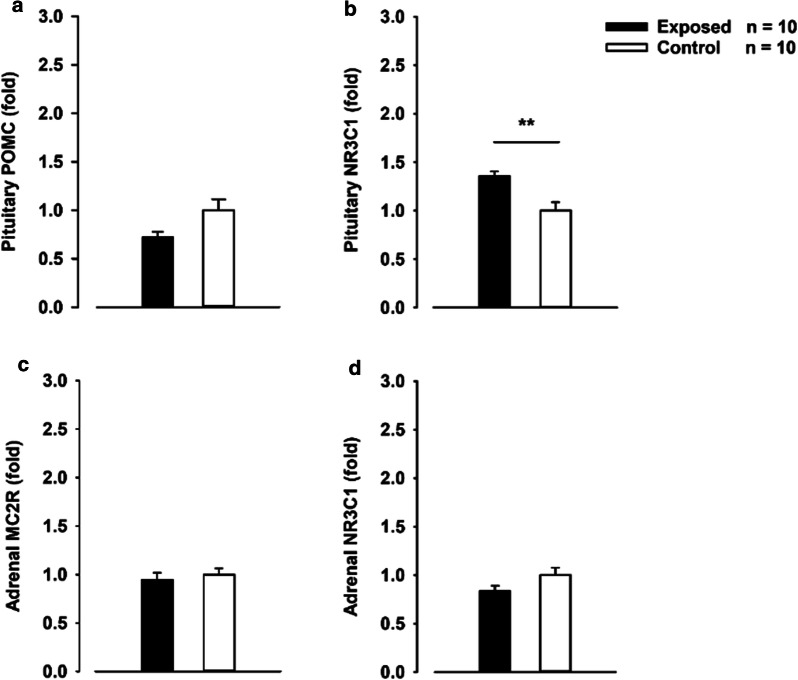
The pituitary- and adrenal- gland. **a**, **b** Fold expression of adrenocorticotropic hormone (ACTH) precursor POMC, and glucocorticoid receptor (Nr3C1) in the pituitary gland of stress-exposed rats versus control rats, p = 0.003. **c, d** Fold expression of melanocortin type-2 receptor (MC2R) and Nr3C1 in the left adrenal gland of stress-exposed rats versus control rats. The data were normalized to β-actin and then to baseline. *p < 0.05, **p < 0.01, Students t-test

The stress exposure did not result in any clear changes of pro-opiomelanocortin (POMC) (Fig. 3a), but showed a significant increase in the expression of NR3C1 in the pituitary gland (Fig. 3b). The exposure did not alter adrenal gland expression of MC2R (ACTH receptor) or NR3C1 (Fig. 3c, d) nor the NE or CORT levels in plasma (Additional file 1: Fig. S2a, b).

### Enrichment of splenic myeloid cells

**Fig. 4:**
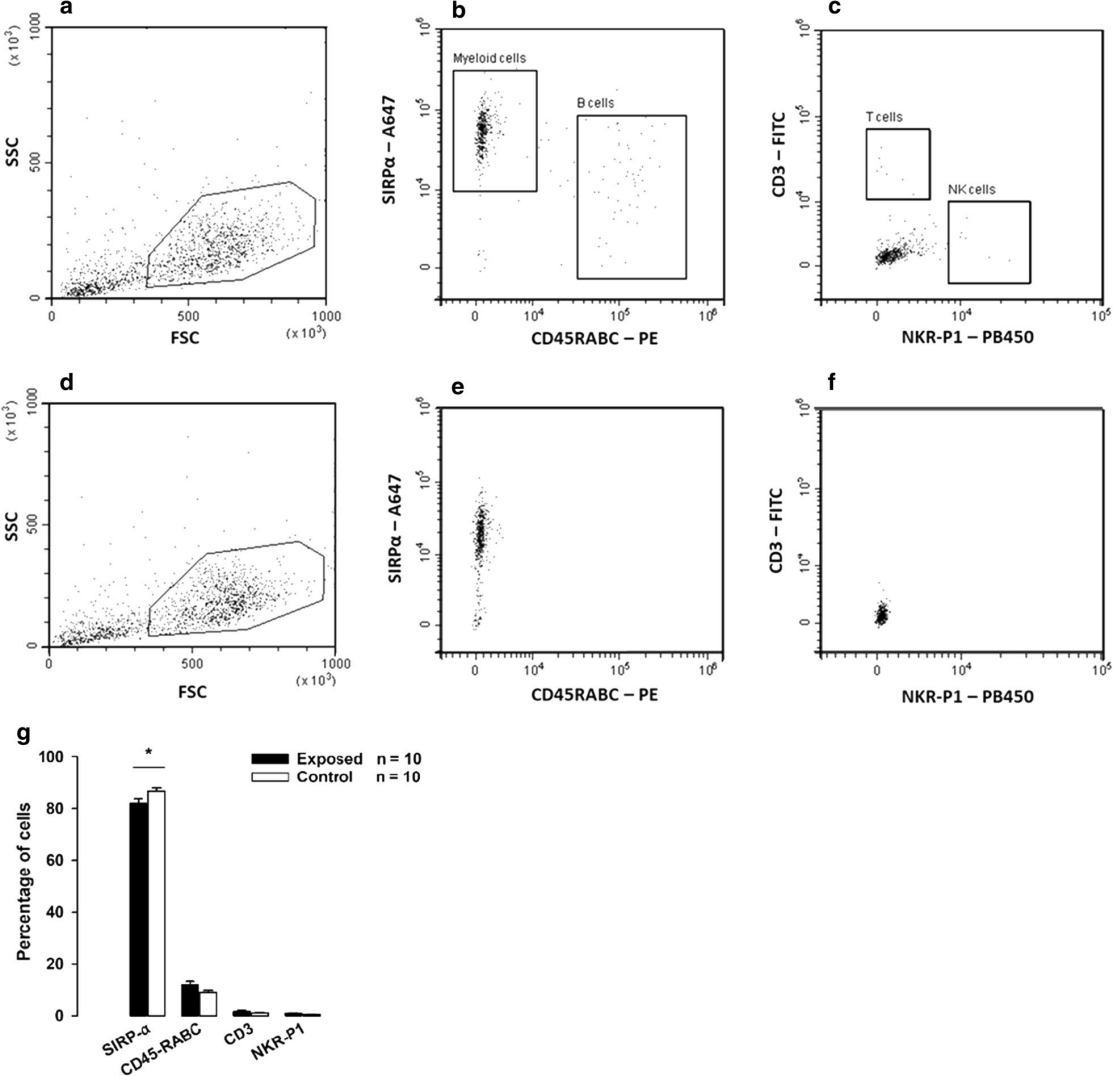
Enrichment of myeloid cells from the spleen. **a** Living cells were included in a polygon gate, whereas the remaining cells (mostly dead and apoptotic cells) were excluded (SSC v FSC). Gated cells (exclusion of the negative population, see Additional file 1: Fig. S3) were visualized with the (**b**) SIRP α–A647 v CD45RABC–PE channels and the (**c**) CD3–FITC v NKR-P1–PB450 channels. **d–f** Scatter plots for isotype controls. **g** Following the enrichment procedure, all samples were analyzed by flow cytometry to characterize the final cell suspension. The mean CD3–percentage of SIRP-α positive cells were 81.9% ± 1.73 and 86.6% ± 1.12 for exposed and control, respectively, p = 0.04. The fraction of contaminating cells made up 12.1% ± 1.28 and 8.8% ± 0.77 CD45RABC positive cells, 1.6% ± 0.51 and 1.1% ± 0.14 CD3 positive cells and 0.9% ± 0.14 and 0.5% ± 0.07 NKR-P1 positive cells in the final isolated cell pellet from exposed and control animals, respectively. *p < 0.05, Students t-test

Flow cytometry analysis of the final cell suspension from the enrichment procedure was performed to elucidate the amount of myeloid cells compared to the amount of contaminating cells (Fig. 4a–f, Additional file 1: Fig.S3). The estimated SIRP-α positive fraction was 81.9% ± 1.73 in the exposed group and 86.6% ± 1.12 in the control group. The predominant contaminating cell type was CD45RABC positive cells (most likely B cells). We observed 12.1% ± 1.28 CD45RABC positive cells in the exposed group and 8.82% ± 0.77 CD45RABC positive cells in the control group (Fig. 4g).

### Gene expression in splenic myeloid cells

**Fig. 5:**
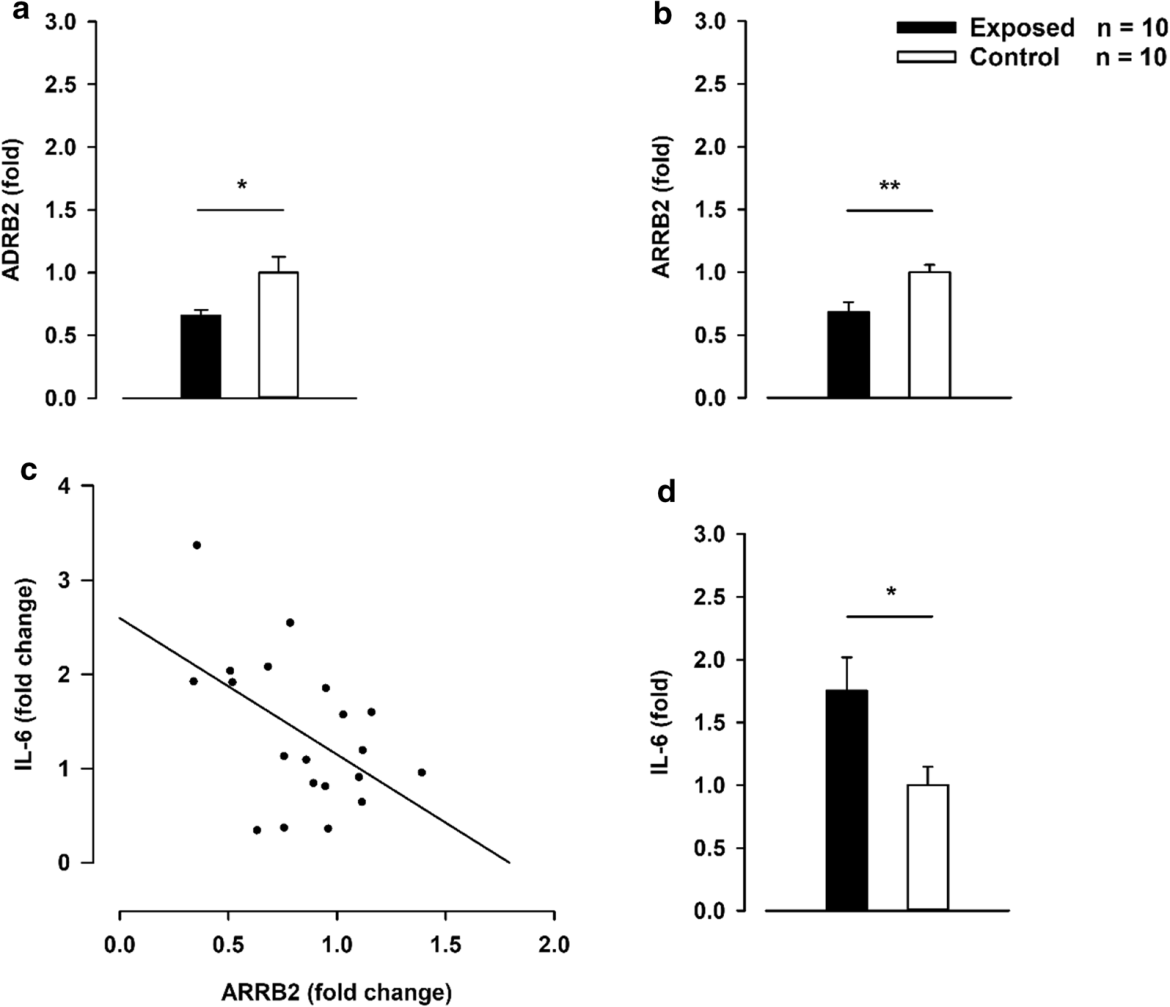
Myeloid cells from the spleen. **a** Fold expression of β2-adrenergic receptor (ADRB2) in the stress-exposed rats versus control rats, p = 0.02. **b** Fold expression of β-arrestin 2 (ARRB2) in stress-exposed rats versus control rats, p = 0.007. **c** The relationship between IL-6 and ARRB2 expression levels, r = 0.509, p = 0.022. **d** Fold expression of interleukin 6 (IL-6) in the stress-exposed rats versus control rats, p = 0.02. All data were normalized to the mean of hypoxanthine phosphoribosyltransferase (HPRT) and protein tyrosine phosphatase receptor type C (PTPRC) and then to the baseline. *p < 0.05, **p < 0.01, Students t-test

The ADRB2 and ARRB2 were both significantly downregulated following 1 week of stress exposure (Fig. 5a, b). In addition, our results showed an increased expression of IL-6 in exposed animals compared to controls and that the IL-6 levels were associated with ARRB2 levels in myeloid cells (Fig. 5c, d). The NR3C1 expression levels revealed no difference in the cell population studied (Additional file 1: Fig. S4).

## Discussion

In the present study, we addressed stress-induced changes in behavior, HPA-axis and immune system. In addition to increased locomotion and reduced weight gain, we observed an increased NR3C1 expression in the pituitary gland after 1 week with social stress. The most robust effects of the stress exposure were, however, seen on isolated splenic immune cells. In these myeloid cells, we observed decreased expression of the ADRB2 and ARRB2, but increased expression of IL-6, the day after the last stress exposure in the paradigm. Moreover, stress exposure induced a downregulation of ARRB2 that was negatively correlated with IL-6. Hence, the present data support the idea that reduced expression of ARRB2 may enhance the translocation of the NF-ĸB to the nucleus and activate the transcription of IL-6.

Earlier findings in murine models indicate that stress-induced physiological changes, including reduced CORT sensitivity in peripheral macrophages, central microglia activation and anxiety-like behavior during stress exposure, may be cycle-dependent, i.e., increases for each stress episode. Previous data also show a stress-induced reduced preference towards sucrose [32] and IL-6 driven energy expenditure affecting gain of body weight [33]. It is therefore tempting to speculate that the observed submissive behavior may involve depression and lack of appetite, but also learned helplessness behavior [34] and enhanced punishment avoidance [35, 36]. In accordance with earlier observations [1, 4, 37], we demonstrated a clear stress-induced decrease in weight gain.

Several studies suggest that the resident-intruder paradigm in mice may cause leukocyte egress from the bone marrow, enhanced recruitment of myeloid cells to the spleen, which in turn may be associated with splenomegaly, i.e., increased spleen weight [21–23, 38]. However, since the enhanced recruitment of myeloid cells to the spleen have been reported to be more pronounced in wounded animals than in those without any injuries [23], the earlier reported stress-induced splenomegaly could be a result of the immunological changes caused by infections etc. Thus, splenomegaly may be a result of physical injuries (wounds) rather than social stress.

In the present study we used rats, not mice. Therefore, it was possible to avoid bites and wounds. The intruder animals in our study were therefore only exposed to social stress, not confounding factors such as physical injuries that could lead to immunological changes caused by infections. Interestingly, the present resident-intruder paradigm in our study, where we used rats, did not change the spleen volume/weight. Therefore, the present study supports the idea that splenomegaly induced by the resident-intruder paradigm may be explained by infections rather than social stress.

Exposure to chronic stress may impair neurogenesis in the prefrontal cortex (PFC) and hippocampus, but have the opposite effect in amygdala [39–41]. Moreover, stress may induce amygdala hyperactivity, increase synaptic connectivity in amygdala [42], and stimulate amygdala-dependent fear learning [43]. Thus, when the exposure to stress persists, the brain seems to switch from slow, attentive PFC regulation to more reflexive responses predominantly controlled by the amygdala and related subcortical structures [44, 45]. Social stress, which involves PFC dysregulation and amygdala hyperactivity, could therefore also promote behavioral changes such as rapid movements observed in the present study.

Previous studies suggest a link between stress-induced migration of leukocytes from the bone marrow and splenomegaly [21–23, 38]. Moreover, the egress of cells from the bone marrow in this process may be controlled by NE/E [22] and CORTs [21]. However, previous data also show that enhanced myeloid recruitment to the spleen could be caused by minor infections following wounds [23]. Thus, whether or not social stress alone is enough to induce splenomegaly may be debated. Our data did not support any clear stress-induced change in spleen weight.

Stress-induced mononuclear cell migration, pro-inflammatory activation, and anxiety-like behavior seem to be catecholamine-dependent [25]. Thus, stress may involve activation of the G protein-coupled adrenergic receptors on leukocytes [46]. Furthermore, earlier findings suggest that NE and/or E activation of ADRB2s may induce the expression of pro-inflammatory cytokines through ERK1/2 and MAPK-dependent mechanisms [27]. In addition to G proteins, cytoplasmic adaptor molecules such as ARRB2 may interact with the ADRB2, conveying signals of anti-inflammatory origin by inhibiting NF-ĸB nuclear translocation [29]. However, PKA- and cAMP-dependent suppression of NF-ĸB can also be induced by ADRB2 signaling.

Interestingly, our data demonstrated reduced ADRB2 and ARRB2 mRNA levels accompanied by increased mRNA levels of IL-6 in the isolated splenic myeloid cells of the stress-exposed rats. It seems plausible that repeated or persistent NE exposure may cause ADRB2 desensitization [47], which is associated with downregulation of ARRB2 [48]. Reduced levels of ARRB2 may result in increased nuclear translocation and transcriptional activity of NF-ĸB. Since NF-ĸB may bind to the IL-6 promoter [49] for review see [50], it seems reasonable to believe that the expression of IL-6 is controlled by the transcription factor NF-ĸB through a promoter binding mechanism [51, 52]. It is tempting to speculate that stress-induced upregulation of IL-6 is a result of reduced ARRB2.

The functional diversity of IL-6 may be reflected through its activation of glycoprotein 130 (gp130) and STAT [53] signal transduction. The ubiquitous expression of gp130 allows for a wide range of actions for the cytokines that utilize this pathway [54]. Signal transduction via gp130 has the capacity to suppress innate immune responses [55] and promote adaptive immunity by lymphocyte trafficking [56]. IL-6 is a key mediator in T cell infiltration of tissue and in the neutrophil to mononuclear cell switch in leukocyte recruitment pattern [57, 58]. Moreover, previous data show that this cytokine is essential for differentiation of naïve T cells and B cells into effector cells [59–61]. In addition, IL-6 production and secretion from splenic myeloid cells may act in an autocrine fashion [53]. Thus, stress-induced splenic upregulation of IL-6 and IL-6 downstream processes may be important for the transition from the acute to persistent immune activation.

## Conclusion

Taken together, our results suggest that the experience of 1 week of repeated social defeat in rats is a potent stressor that triggers prolonged myeloid inflammatory changes in lymphoid tissues such as the spleen. We believe our results demonstrate neuroendocrine and immunological changes caused by social stress only, not confounding factors such as physical injuries and infections often seen in mice. This shows that the inflammatory effect of such social stress may be stronger than previously assumed. The role of this mechanism following exposure to social stress in humans remains to be investigated.

## Supplementary information

**Additional file 1: Figure S1.** The test used to assess behavior. **Figure S2.** Plasma**. Figure S3.** Gating strategy to assess purity of enriched myeloid cell population from rat spleen. **Figure S4.** Myeloid cells from the spleen. **Table S1.** Two separate mixes of fluorochrome-conjugated monoclonal antibodies for test and control. The two mixes were separately added to a small fraction of the final cell suspension.

## Data Availability

The datasets supporting the conclusions of this article are included within the article and its additional files.

## Abbreviations

ADRB2: β2-adrenergic receptor
ARRB2: β-arrestin-2
CORT: Corticosterone
CRH: Corticotropin-releasing hormone
E: Epinephrine
ERK: Extracellular signal-regulated kinases
GC: Glucocorticoid
GM-CSF: Granulocyte-macrophage colony-stimulating factor
HPA: Hypothalamic–pituitary–adrenal
IL-6: Interleukin 6
IL-1β: Interleukin 1 β
LC: Locus coreleus
MAPK: Mitogen activated protein kinase
MCP-1: Monocyte chemoattractant protein-1
NE: Noradrenaline
NFKB: Nuclear factor kappa-lit-chain-enhancer of activated B cells
NR3C1: Nuclear receptor subfamily 3, group c, member 1
PFC: Prefrontal cortex
POMC: Pro-opiomelanocortin
PVN: Paraventricular nucleus
SNS: Sympathetic nervous system
STAT: Signal transducer and activator of transcription
