# Supplementary material for: Repeated social defeat promotes persistent inflammatory changes in splenic myeloid cells; decreased expression of β-arrestin-2 (ARRB2) and increased expression of interleukin-6 (IL-6)
Source: BMC Neurosci. 2020 May 29;21:25. doi: 10.1186/s12868-020-00574-4 (PMC7260804; doi:10.1186/s12868-020-00574-4)
Supplement: Supplementary file 1 — Additional file 1: Figure S1. The test used to assess behavior. Figure S2. Plasma. Figure S3. Gating strategy to assess purity of enriched myeloid cell population from rat spleen. Figure S4. Myeloid cells from the spleen. Table S1. Two separate mixes of fluorochrome-conjugated monoclonal antibodies for test and control. The two mixes were separately added to a small fraction of the final cell suspension. [file 12868_2020_574_MOESM1_ESM.docx]

**Additional figures**

**Additional file 1: figure S1**

| **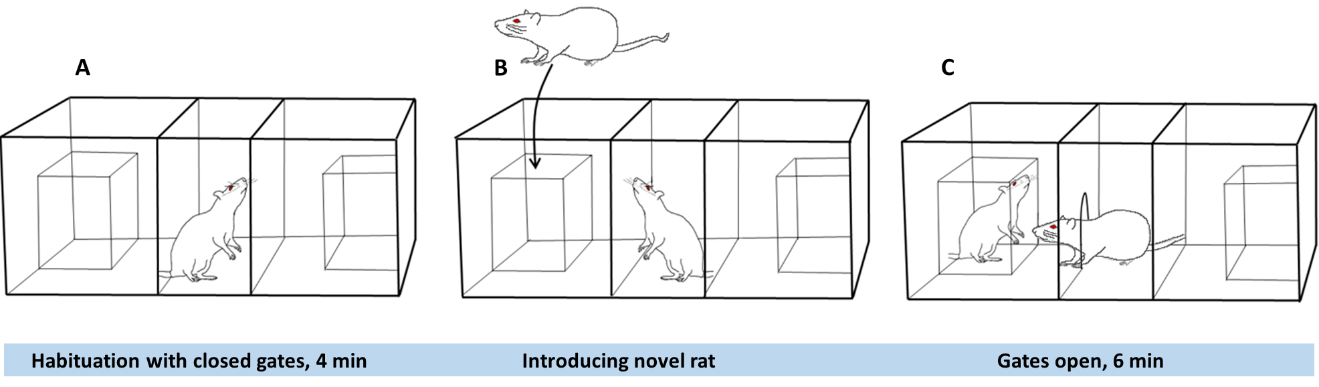** |
| --- |

**Additional file 1: figure S2**

| **** |
| --- |

**Additional file 1: figure S3**

| 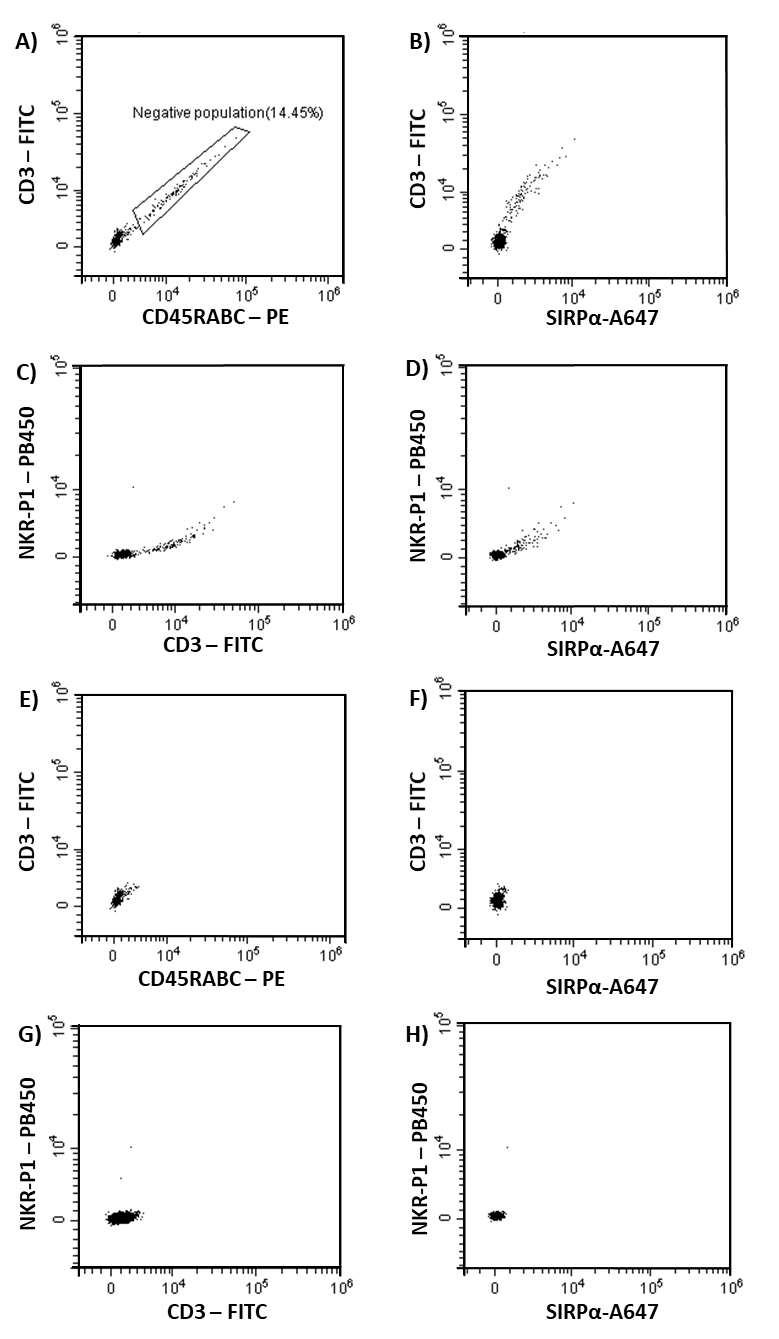 |
| --- |

| **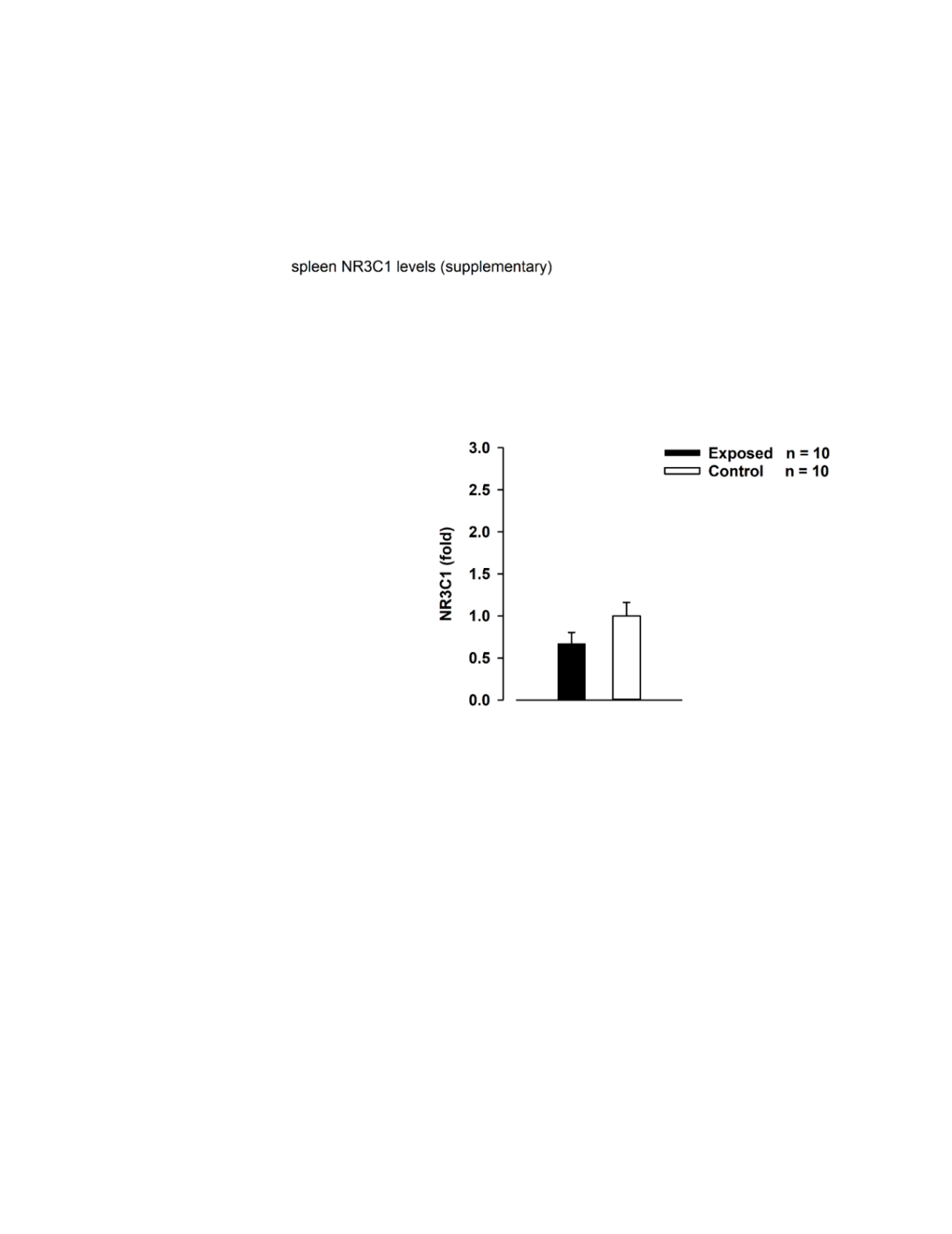** |
| --- |

**Additional file 1: figure S4**

**Additional file 1: figure legends**

**Additional file 1: figure S1. The test used to assess behavior.** The test arena consisted of a box separated into three chambers by gated plastic walls. **(A)** First, the test rat was placed in the middle chamber for four minutes. **(B)** Next, a novel rat of the same strain was placed in a small wire-like container in one of the flanking compartments. **(C)** Third, the gates were open for six minutes, allowing the test rat to explore all parts of the box.

**Additional file 1: figure S2. Plasma. (A)** Plasma noradrenaline (NE) levels and **(B)** corticosterone (CORT) levels in stress-exposed rats versus control rats.

**Additional file 1: figure S3. Gating strategy to assess purity of enriched myeloid cell population from rat spleen.** FSC x SSC gating (not shown) on unstained sample was used to exclude dead cells and cell debris. **(A-D)** Living cells were plotted with the CD3 – FITC v CD45RABC – PE channels, CD3 – FITC v SIRPα-A647 channels, NKR-P1 – PB450 v CD3 – FITC channels and the NKR-P1-PB450 v SIRPα-A647 channels. Negative population included in the gate (shown in Supplementary fig 3A) was positive for both B- and T-cell markers. Theoretically, this population does not exist. The negative population might include sticky myeloid cells, because of unspecific antibody binding due to poor Fc receptor blocking, or Miltenyi microbeads used during the magnetic separation in the myeloid cell enrichment protocol. Inclusion of the negative population may result in addition of false positives to the analyses. **(E-H)** Hence, the negative population was excluded from the rest of the flow cytometry analyses.

**Additional file 1: figure S4. Myeloid cells from the spleen.** Fold expression of Nr3C1 in stress-exposed rats versus control rats. The data was normalized to the mean of phosphoribosyltransferase (HPRT) and protein tyrosine phosphatase receptor type C (PTPRC) and then to the baseline.

**Additional file 1: Table S1**

| **Mix 1 (test)** |  |  |  |
| --- | --- | --- | --- |
| **Target** | **Antigens** | **Antibody-conjugate** | **Final conc.** |
| Myeloid cells | CD172a/ SIRP-α | OX41-biotin | 2 µg/ mL |
| B cells | CD45RABC | OX33-PE | 2 µg/ mL |
| T cells | CD3 | G4.18 - FITC | 2 µg/ mL |
| NK cells | NKR-P1 | 3.2.3 – Pacific blue | 2 µg/ mL |

**Mix 2 (isotype control)**

|  |  | **Isotype Ab-conjugate** | **Final conc.** |
| --- | --- | --- | --- |
|  |  | IgG1-biotin | 2 µg/ mL |
|  |  | IgG1-PE | 2 µg/ mL |
|  |  | IgG3 - FITC | 2 µg/ mL |
|  |  | IgG1 – Pacific blue | 2 µg/ mL |

**Additional file 1: Table S1** Two separate mixes of fluorochrome-conjugated monoclonal antibodies for test and control. The two mixes were separately added to a small fraction of the final cell suspension.
